# A Search for the Optimum Lithium Rich Layered Metal Oxide Cathode Material for Li-Ion Batteries

**DOI:** 10.1149/2.0481507jes

**Published:** 2015-04-09

**Authors:** Mehmet Nurullah Ates, Sanjeev Mukerjee, K. M. Abraham

**Affiliations:** Center for Renewable Energy Technology, Department of Chemistry and Chemical Biology, Northeastern University, Boston, Massachusetts 02115, USA

## Abstract

We report the results of a comprehensive study of the relationship between electrochemical performance in Li cells and chemical composition of a series of Li rich layered metal oxides of the general formula xLi_2_MnO_3_ · (1-x)LiMn_0.33_Ni_0.33_Co_0.33_O_2_ in which x = 0,1, 0.2, 0,3, 0.5 or 0.7, synthesized using the same method. In order to identify the cathode material having the optimum Li cell performance we first varied the ratio between Li_2_MnO_3_ and LiMO_2_ segments of the composite oxides while maintaining the same metal ratio residing within their LiMO_2_ portions. The materials with the overall composition 0.5Li_2_MnO_3_ · 0.5LiMO_2_ containing 0.5 mole of Li_2_MnO_3_ per mole of the composite metal oxide were found to be the optimum in terms of electrochemical performance. The electrochemical properties of these materials were further tuned by changing the relative amounts of Mn, Ni and Co in the LiMO_2_ segment to produce xLi_2_MnO_3_ · (1-x)LiMn_0.50_Ni_0.35_Co_0.15_O_2_ with enhanced capacities and rate capabilities. The rate capability of the lithium rich compound in which x = 0.3 was further increased by preparing electrodes with about 2 weight-percent multiwall carbon nanotube in the electrode. Lithium cells prepared with such electrodes were cycled at the 4C rate with little fade in capacity for over one hundred cycles.

The energy densities of Li-ion batteries are limited by the capacities of cathode materials. The highest energy density commercially available Li-ion batteries utilize graphite anodes with a capacity of about 370 mAh/gram, and LiNi_0.8_Co_0.15_Al_0.05_O_2_ (NCA) cathodes exhibiting approximately190 mAh/gram capacity. The latter corresponds to the reversible charge/discharge cycling of 0.7 Li per transition metal, the highest capacity achievable in these and related layered metal dioxide (LiMO_2_) congeners of LiCoO_2_. The reversible cycling of one Li per transition metal is not possible in these positive electrode materials because they cannot be charged to a voltage necessary to fully extract all the Li in LiMO_2_, in which M = Ni and Co, due to the instability of the fully delithiated metal dioxides NiO_2_ and CoO_2_ containing Ni and Co in the 4^+^ oxidation state.

The Li rich transition metal dioxides of the general formula xLi_2_MnO_3_ · (1-x)LiMn_a_Ni_b_Co_c_O_2_ are promising class of cathode materials capable of delivering discharge capacities > 250 mAh/gram or the transfer of nearly one Li per transition metal. They exhibit such high capacities once the materials are charged initially to a voltage between 4.6 and 4.9 V versus Li/Li^+^.^1–4^ Their high capacity stem from the activation of Li_2_MnO_3_ in the first charge beginning at approximately 4.5 V in a Li cell. This process encompasses a very complex mechanism triggering transition metal (TM) migration into vacant Li and/or TM sites leading to voltage hysteresis in the following cycles. Although the origin of the voltage fade is still under debate,^5–8^ data we obtained with 0.3Li_2_MnO_3_.0.7LiNi_0.5_Co_0.5_O_2_ in which Mn was absent in the LiMO_2_ segment^9^ indicated unambiguously that the latter Mn was responsible for the voltage fade. This layered composite metal oxide showed little voltage fade with cycling at 50°C at which electrodes containing Mn in both segments of the layered composite metal oxide showed significant voltage fade. All of the studies previous to our work were done with materials containing Mn in both segments of the layered composite so that the results from those investigations were not unambiguous. Stable voltage responses and good capacity retentions of cathode materials are crucial to obtain high energy and power densities and the long cycle life needed for Li-ion batteries suitable for portable consumer products and electric vehicle propulsion. A great deal of recent studies has been aimed at mitigating or arresting the voltage fade and power loss of these materials with repeated charge/discharge cycling. One way to suppress the voltage fade is through alkali metal cation (Na^+^, K^+^) doping^10–12^ in which the cation plays a pillaring role within the structure with the result of arresting the transition metal migration and voltage fade with cycling. In addition, coating the cathode material was found to be effective in reducing the voltage decay^13,14^ by suppressing oxygen vacancies created during the first cycle, and mitigating side reactions at high potentials. Also, it has been shown^9,15^ that the amount of Mn in these classes of material have a direct impact on the layered to spinel structural conversion accompanying the cycling-related voltage decay. However, none of these structural and surface modifications have been able to fully overcome the performance deficiencies of these cathode materials including the large irreversible capacity loss (ICL) in the first cycle,^16^ voltage hysteresis and structural transformation that accompany charge/discharge cycling,^6^ and the inability to maintain stable capacity over hundreds of cycles^17^ at either low or high rates.

Although a number of studies^5,18–24^ have attempted to unravel the root causes of these issues they utilized materials containing different types of transition metals and/or ratios of transition metals prepared via different synthesis methods without the ability to provide a systematic understanding of the relationship between cathode materials structure and electrochemistry in Li-ion cells. The conclusions of each work, therefore, might not necessarily be relevant to each other and could mislead the battery community. In an effort to systematically elucidate the structure-electrochemistry relationships in these high capacity cathode materials, we have prepared a series of materials of the general formula xLi_2_MnO_3_ · (1-x)LiMn_a_Ni_b_Co_c_O_2_ using the same synthesis method, and performed a detailed study of the relationship among their material composition, structure and electrochemical performance as cathodes in Li-ion batteries.

We synthesized a series of Li rich layered metal oxide cathode materials using the co-precipitation technique. In these materials Li and transition metal oxide contents were varied systematically in order to assess how the relative amounts of Li, Mn, Co and Ni in the mixed metal oxides affect their capacity and rechargeability, and influence capacity fade and voltage slippage as a function of charge/discharge cycling of Li cells at various rates. We eliminated property differences due to synthesis by preparing all of the materials via the same method. In addition, the ratio of Li_2_MnO_3_ to LiMO_2_ in the composite oxide structure was varied to correlate electrochemical performance with oxide structure. To enhance the rate capability of Li rich compounds, we studied the effect on Li cell electrochemistry of addition of multi-walled carbon nanotube (MWCT) to the cathode electrode composition. We employed a multitude of experimental methods including X-ray diffraction (XRD) to identify structural phase information, Field-emission scanning electron microscopy (FESEM) to observe surface features of the synthesized cathode materials, and X-ray absorption spectroscopy (XAS) to study local geometry and valence state variations of transition metals in each material. The electrochemical properties of each material were discerned from cyclic voltammetry (CV) and galvanostatic cycling data in Li cells.

## Experimental

### Preparations of materials

A series of Li rich layered metal oxide cathode materials of the general formula xLi_2_MnO_3_ · (1-x) LiMn_a_Ni_b_Co_c_O_2_ were synthesized using the conventional co-precipitation method followed by a high temperature treatment.^1,10,15^ Briefly, appropriate amounts of nitrate salts of the transition metals (Mn, Ni and Co) were dissolved in deionized water. The dissolved metal nitrates were then slowly dropped into a mildly alkaline solution of LiOH.H_2_O through a burette. The time for adding the metal nitrates into the alkaline solution was set to be around 1 h. The pH of the solution was maintained around 12 by monitoring with a SypmHony model SP70P pH meter. When co-precipitation was completed, the mixed metal hydroxide precipitate was washed with copious deionized water three times in order to remove any residues of alkali metal nitrates. After drying the mixed metal hydroxide precipitate in a vacuum oven, it was thoroughly mixed with an appropriate amount of LiOH.H_2_O and calcined first at 480°C for 3 h, then grounded, pelletized and calcined again at 900°C for another 3 h under air to obtain the Li rich layered metal oxide powders. In order to obtain a Ni^4+^ reference material for XAS experiments we also synthesized LiNi_0.85_Co_0.15_O_2_ which produced Ni^4+^ when it was charged to 5.1 V. Details of this synthesis can be found in our previous publication^10^and elsewhere.^25^

### Materials characterizations

The crystalline phase of each Li rich cathode powder was characterized by Rigaku Ultima IV X-ray diffractometer using CuKα radiation. The scan range was set between 10–80 2θ degrees for each measurement. The crystal structures of all as-synthesized powders were processed with the aid of PDXL software, provided by Rigaku Corporation. Unit cell visualization was drawn with VESTA software. The surface feature of each powder was investigated with a Hitachi S-4800 Field Emission Scanning Electron Microscopy (FESEM). To unravel local geometry and valence states of each transition metal in the composite metal oxide cathode, we ran X-ray absorption spectra at beam line X-3A and X18-A of the National Synchrotron Light Source (NSLS-I) located at Brookhaven National Laboratory. XAS experiments were performed in ex-situ mode using electrodes extracted from coin-cells. The electrodes were sealed with Kapton tape and stored in glass vials followed by packing in moisture impermeable aluminized bags in Ar filled glove box before transporting to NSLS-I. Each raw scan was calibrated, normalized and aligned with respect to reference foils through Artemis software.^26^

### Electrochemical tests

Galvanostatic cycling tests were performed at room temperature using coin cells. The cells were cycled between 2 and 4.9 V unless otherwise stated. For the purpose of cycle life and rate capability assessments, an Arbin BTZ2000 model cycler was employed. The cathode for a coin cell was prepared in four steps: i-) 10 wt% polyvinylidene fluoride (PVDF) was dissolved in an NMP-containing vial mounted in a sonicator bath, ii-) 80 wt% of cathode active material and 10 wt% of Super P carbon were intimately mixed and dropped into the NMP mixture, iii-) the resulting ink was coated on Al foil substrate via doctor-blade to control the thickness of the electrodes, and finally iv-) the electrodes were vacuum dried under 120°C overnight. The electrolyte employed in this work was obtained by dissolving 1 M LiPF_6_ in a 1:1.2 EC/DMC mixture. The *C* rates were calculated using the same theoretical capacity of 280 mAh/g based on 1 Li per MO_2_ for all of the Li rich metal oxide cathode materials studied. For more details on the electrochemical test conditions, readers are referred to our previous publication.^10^Cyclic voltammograms of the Li cells were recorded at room temperature in the range of 2–4.9 V at a 50 μV/s scan rate using VoltaLab PGZ402 model potentiostat.

## Results and Discussion

Our investigations to unravel the composition, structure and electrochemistry relationships in Li rich layered metal oxides proceeded along three avenues. First we synthesized a series of materials of the formula xLi_2_MnO_3_ · (1-x)LiMn_0.33_Ni_0.33_Co_0.33_O_2_ (0.1≤x≤0.7) and studied their structure and electrochemistry in detail. We further explored the 0.5Li_2_MnO_3_ · 0.5LiMn_a_Ni_b_Co_c_O_2_ composition by preparing two materials in which the amounts of Mn, Ni and Co in the LiMO_2_ segment were varied since this composite with 0.5 mole of Li_2_MnO_3_ was found to be the optimum material in terms of electrochemical performance. Finally, our efforts were directed toward the enhancement of rate capabilities of the Li rich layered metal oxide cathode material by MWCT addition to the electrode composition.

### The effect of the ratio of Li_2_MnO_3_ to LiMO_2_ on structure and elec-trochemistry of xLi_2_MnO_3_ · (1-x)LiMn_0.33_Ni_0.33_Co_0.33_O_2_

**Figure 1:**
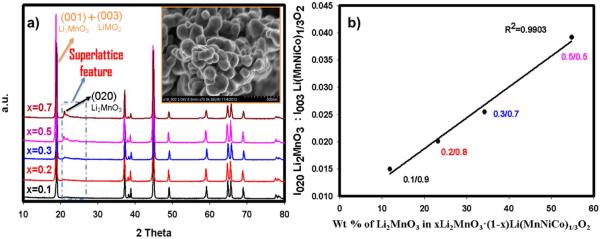
a-) XRD patterns for xLi_2_MnO_3_ · (1-x)LiMn_0.33_Ni_0.33_Co_0.33_O_2_ where x ranges from 0.1 to 0.7. FESEM image shows particle sizes approximately 250 nm. b-) a plot of the weight percentage of xLi_2_MnO_3_ · (1-x)LiMn_0.33_Ni_0.33_Co_0.33_O_2_ as a function of the ratio of first peak intensities of Li_2_MnO_3_(020) to LiMO_2_ (003) planes.

The various metal oxides we prepared and characterized and their first charge and the subsequent discharge capacities measured are presented in Table I. The XRD patterns allow a visual inspection of the effect of Li_2_MnO_3_ on the crystal structure of each material. Figure 1a displays the XRD patterns for five xLi_2_MnO_3_ · (1-x)LiMn_0.33_Ni_0.33_Co_0.33_O_2_ (0.1≤x≤0.7) cathodes, all synthesized through the co-precipitation method. All of the materials presented in the Figure 1a possess the same 1:1:1 ratio of Mn:Ni:Co in the LiMO_2_ segment with only the Li_2_MnO_3_ to LiMO_2_ ratio varying from one material to the other. This allows us to understand how Li_2_MnO_3_ contribute to the first charge capacity, the reversible capacities following the first charge, the change in the charge/discharge voltage profiles from the first to the subsequent cycles, and the decay in the discharge voltage accompanying repeated charge/discharge cycling. Figure 1a shows that as the amount of Li_2_MnO_3_ increases, the corresponding superlattice feature appearing between about 20 and 26 2θ degrees, highlighted in the rectangle, also becomes more visible. A plot of the ratio of I(020), which solely belongs to Li_2_MnO_3_, to I(003)_LiMO2_ and/or I(001)_Li2MnO3_ versus the actual weight percentage of Li_2_MnO_3_ in the series of xLi_2_MnO_3_ · (1-x)LiMn_0.33_Ni_0.33_Co_0.33_O_2_ (0.1≤x≤0.5) cathodes, displayed in Figure 1b, approximately intersects through the origin manifesting excellent correlation between the weight ratio of Li_2_ MnO_3_ and its respective feature in the XRD pattern.^15^Surface morphologies of the Li rich powders were investigated and a representative FESEM image corresponding to 0.2Li_2_MnO_3_ · 0.8LiMn_0.33_Ni_0.33_Co_0.33_O_2_ is shown as the inset of Figure 1a. The nano particles comprising the material are agglomerated to form larger 200–400 nm grains. No significant difference was detected in the morphologies of the different cathode materials. The higher Mn resulted in materials with a brown color while higher Co causes the final materials to acquire a black color.

**Figure 2:**
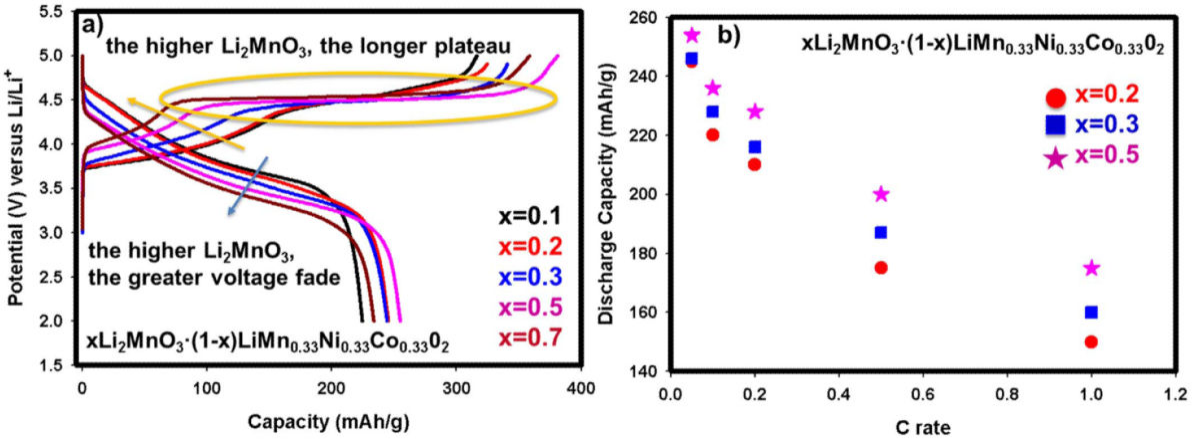
a-) The first charge-discharge profiles for xLi_2_MnO_3_ · (1-x)LiMn_0.33_Ni_0.33_Co_0.33_O_2_ (0.1≤x≤0.7) at C/20 rate between 2 and 4.9 V at room temperature. b-) rate capability at room temperature for materials where x = 0.2, 0.3 and 0.5.

The 1^st^ electrochemical charge and discharge profiles for Li rich cathodes are shown in Figure 2a. The length of plateau region in the first charge was found to correlate with the amount of Li_2_MnO_3_ since 0.7Li_2_MnO_3_ · 0.3LiMn_0.33_Ni_0.33_Co_0.33_O_2_ with the greatest amount of Li_2_MnO_3_ has the longest 4.6 V plateau region in the first charge. The sloping first discharge voltage of the same material, however, has the highest hysteresis in addition to inferior discharge capacity compared to the other materials presented in Figure 2a. One can infer from these results that a very high Li_2_MnO_3_ results in low discharge voltage slopes and low specific discharge capacities. The materials with x = 0.2, 0.3 and 0.5 are particularly appealing based on their high capacities obtained in the first cycle. Nevertheless, in terms of capacity retentions during further cycling and rate capabilities, 0.5Li_2_MnO_3_ · 0.5LiMn_0.33_Ni_0.33_Co_0.33_O_2_ (x = 0.5) was found to be the optimum composite oxide among the various Li rich materials shown in Figure 2a. The graph depicting the rate capabili-ties of the x = 0.2, 0.3 and 0.5 compounds are shown in Figure 2b. Previous studies had demonstrated that Li_2_MnO_3_ alone possess extremely low rate capability and cycling ability.^27^Therefore, it is crucial to create enough MnO_2_ as a reservoir for Li^+^ that cannot be hosted by LiMO_2_ after de-intercalation. We showed here that the composite, having 50% of Li_2_MnO_3_ and 50% of LiMO_2_ (i.e. x = 0.5), host approximately 1 Li per metal redox reaction leading to optimum capacity.

**Figure 3:**
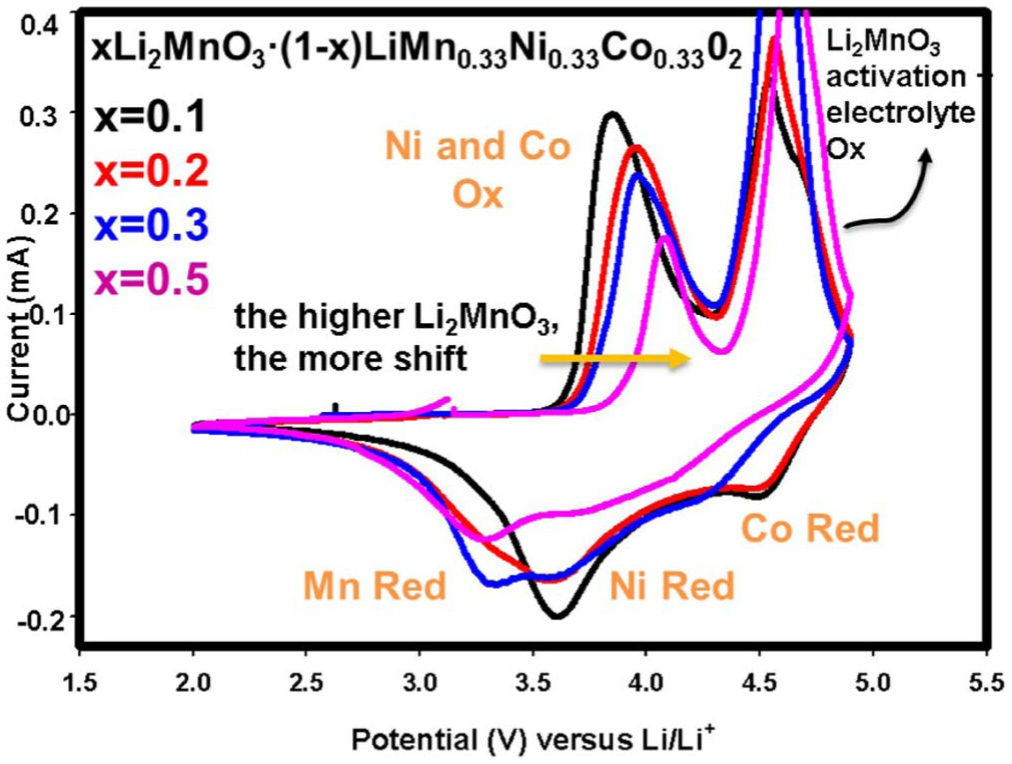
The first cyclic voltammograms run at 50 μV/s for xLi_2_MnO_3_ · (1-x)LiMn_0.33_Ni_0.33_Co_0.33_O_2_ (0.1≤x≤0.5) scanned between 2 and 4.9 V at room temperature.

Cyclic voltammetry (CV) scans were performed with the materials in order to gain further insight in to the redox behavior of the transition metals in the composite oxides. Figure 3 displays the CV profiles of four of the composite oxides xLi_2_MnO_3_ · (1-x)LiMn_0.33_Ni_0.33_Co_0.33_O_2_ with x = 0.1, 0.2, 0.3, and 0.5 where the shape of redox behavior and redox peak apexes are notably different. First of all, as the amount of Li_2_MnO_3_ increases, a clear trend is observed for the first oxidation peak at around 4 V which is probably due to the concurrent oxidations of Ni and Co. The higher amount of Li_2_MnO_3_ caused this peak to move toward higher potentials. Thus for 0.5Li_2_MnO_3_ · 0.5LiMn_0.33_Ni_0.33_Co_0.33_O_2_ the oxidation takes place slightly above 4 V whereas the same process occurs at around 3.8 V for 0.1Li_2_MnO_3_ · 0.9LiMn_0.33_Ni_0.33_Co_0.33_O_2_. This is an interesting finding since Ni and Co do not exist in the Li_2_MnO_3_ segment; rather they reside within the LiMO_2_ fraction. However, the trend observed in the first oxidation peak with the amount of Li_2_MnO_3_ present indicates that the latter directly influences the redox behavior of LiMO_2_ fraction. This suggests structural correlation between the two segments in the composite oxide at the atomic level. In other words, the original Li_2_MnO_3_ affects the redox nature of LiMO_2_. During the first discharge, while materials having x = 0.1 and 0.2 compositions have sharp peak features,^15^cathodes comprised of higher amount of Li_2_MnO_3_ yielded broader peaks suggesting that structural rearrangements involving metal migration occurs in x = 0.3 and 0.5 compositions at higher degrees of Li extraction. The former advocates homogeneous reaction kinetics while the latter suggest more likely heterogeneous reaction mechanism. A lower amount of Li_2_MnO_3_ appears to trigger electrochemical reaction primarily in single-phase LiMO_2_ while a higher amount of Li_2_MnO_3_ stimulates involvement of a two-phase phenomenon where Li_2_MnO_3_ and LiMO_2_ are jointly contributing to the redox capacities of the composite cathode.

**Figure 4:**
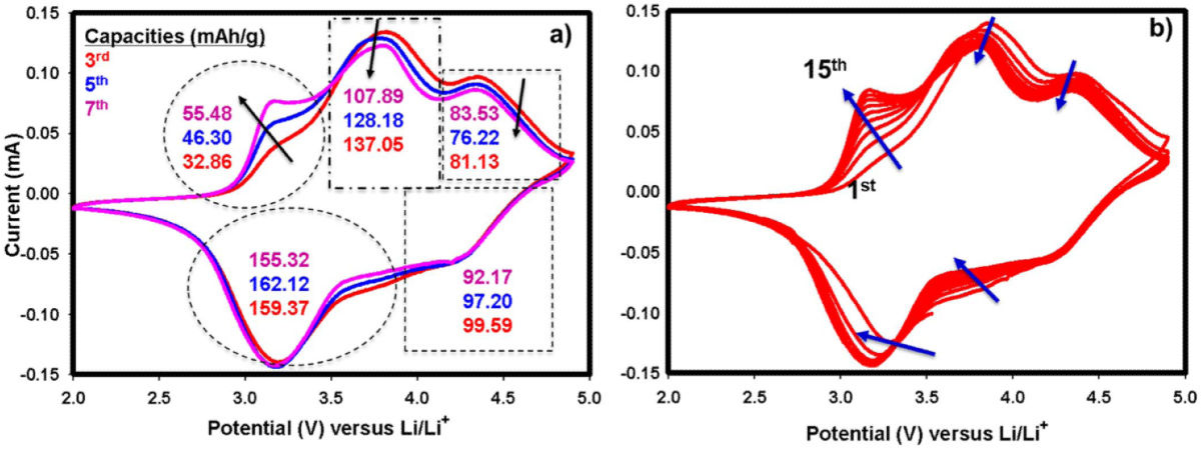
a-) The initial (3^rd^, 5^th^ , 7^th^ ) CV profiles of 0.5Li_2_MnO_3_ · 0.5LiMn_0.33_Ni_0.33_Co_0.33_O_2_ compound along with calculated capacity contributions for each redox reactions. b-) long term CV profiles for the same material and cell presented in Figure 4a. The cell was scanned at sweep rate of 50 μV/s between 2 and 4.9 V at room temperature.

**Figure 5:**
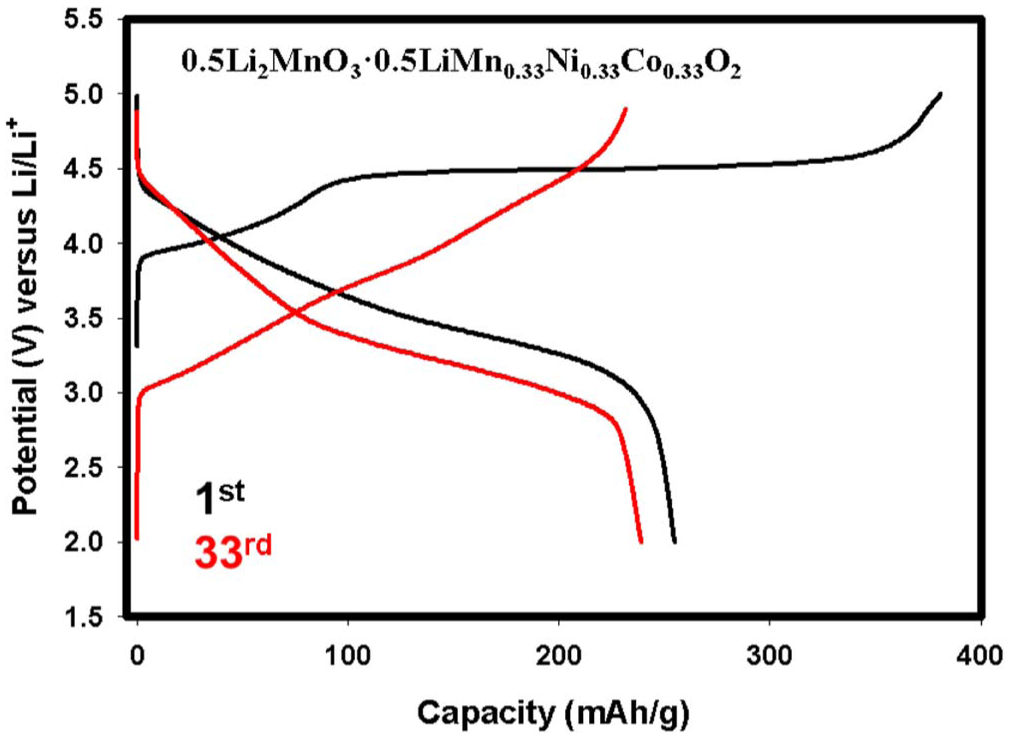
Charge-discharge profiles for the 1^st^ and 33^rd^ cycles of 0.5Li_2_MnO_3_ · 0.5LiMn_0.33_Ni_0.33_Co_0.33_O_2_ at room temperature showing voltage fade.

We have chosen 0.5Li_2_MnO_3_ · 0.5LiMn_0.33_Ni_0.33_Co_0.33_O_2_ for repeated CV cycling while noting that the conclusions of each materials’CV would have been interpreted differently due to different kinetic mechanisms as explained above. Figure 4a shows the first 7 cycles, after the activation of Li_2_MnO_3_, and the capacities obtained in each cycle at the different peaks corresponding to the different TM oxidation and reduction reactions. For example oxidation peak around 3.2 V corresponding to Mn from MnO_2_, is initially ill-defined, but becomes more pronounced as cycling progresses. This behavior is due to the dynamic activation process of Li_2_MnO_3_. After activation, it yields MnO_2_ and this product begins to host Li as cycling continues and forms a more perfect LiMO_2_ layered phase in conjunction with other transition metals. Although Ni and Co cannot be differentiated in CV, our recent results of XAS^10^and previous reports^28^have shown that part of Co oxidation takes place after 4.4 V. Having said that, one can assume that the oxidation peak around 3.8 V most probably pertains to Ni^2+^to Ni^4+^ oxidation. This peak and its capacity contribution, however, starts to decrease as cycling proceeds. The oxidation peak at around 4.5 V, corresponding to Co^3+^to Co^4+^ oxidation, seemingly stabilizes itself in the first few cycles. During the discharge in the first few cycles, there is no significant and discernable changes observed in terms of capacity and voltage hysteresis. One way to determine the accuracy of the CV tests is to calculate the capacity contribution from the CV curves where redox reactions occur. The total discharge capacity in the 3^rd^ cycle calculated from the CV data in Figure 4a equals 259 mAh/g which perfectly matches with the first discharge capacity obtained galvanostatically at low rates shown for the same material (i.e. x = 0.5) in Figure 2a. This further supports the successful Li extraction/insertion reaction in the CV cell. In contrast to this stability, it is found that in the long-run all peak positions and intensities are slightly altering as seen in Figure 4b which presents the continued CV cycling of the cell presented in Figure 4a. During further cycling, the tendency to transform to spinel-like behavior is observed as evidenced by a voltage shift during reduction toward below 3 V,^6,16^ a characteristic feature of LiMn_2_O_4_. This shift can be quickly realized in the galvanostatically charged and discharged sample in Figure 5 in the form of voltage depression and capacity fade. From this Figure, voltage fade is evident from the slopes of the 1^st^ and the 33^rd^ discharges. The peak position of Mn oxidation in Figure 4b is stable; however, as cycling continues the intensity of the Mn oxidation peak increases due to dynamic insertion of Li into MnO_2_, generated during the activation of Li_2_MnO_3_. Relatively small Ni and Co oxidation and their respective intensities decreased as well. Overall, it is clear from these data that stable and reliable voltage responses and redox behavior of each TM could only be obtained after at least 50–60 cycles.

**Figure 6:**
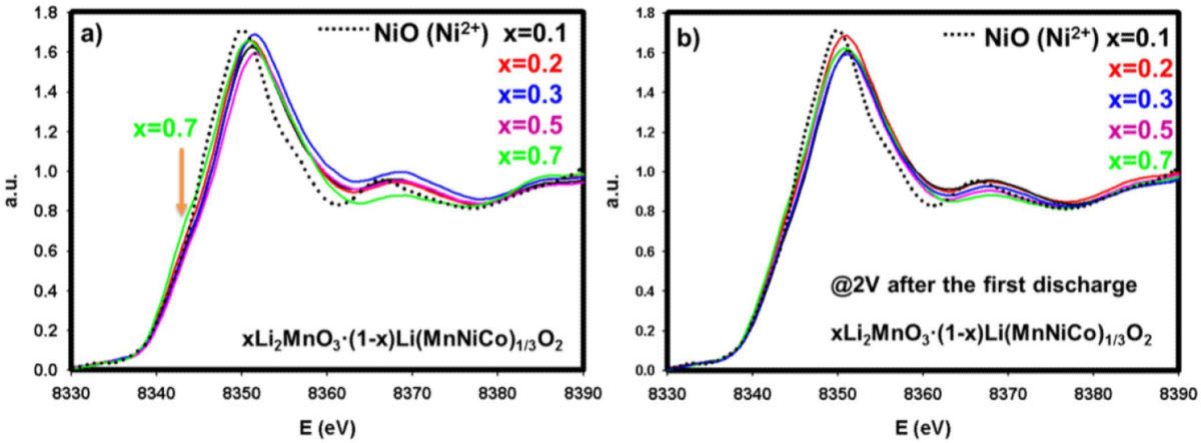
a-) XANES profiles for pristine materials xLi_2_MnO_3_ · (1-x)LiMn_0.33_Ni_0.33_Co_0.33_O_2_ (0.1≤x≤0.7) at Ni-K edge with Ni^2+^reference compound. b-) XANES profiles at Ni-K edge for the same materials after one full cycle, discharged to 2 V.

In an attempt to understand the chemical environment of Ni atom, a major metal contributor to the overall capacity, we performed XAS on Ni-K edge for all materials ranging from x = 0.1 to x = 0.7 in both the pristine states and after discharge to 2 V. Figure 6a displays the Ni-K-edge XANES spectra of the various compounds along with that of NiO (Ni^2+^) reference compound. One discernable feature in this Figure is that the material, coded as x = 0.7, having the highest amount of Li_2_MnO_3_, shows a small shoulder right after 8340 eV. This clearly advocates that the 0.7Li_2_MnO_3_ · 0.3LiMn_0.33_Ni_0.33_Co_0.33_O_2_ compound has different chemical environment for Ni than for the other materials. The threshold of white line, a tool to identify the valence state of TM, slightly differs due to different amount of other transition metals affecting the oxidation state of Ni. All of the materials have an average oxidation state of 2^+^ for the Ni atom. After charging each materials up to 4.9 V and discharging to 2 V, all compounds, shown in Figure 6b, including x = 0.7, resemble each other in terms of XANES shapes. This suggests that after activation of Li_2_MnO_3_, the structure transforms to a regular layered phase grouped as R3m. All materials recovered their oxidation state back to the pristine state after one full cycle.

### The effect of transition metal ratio in 0.5Li_2_MnO_3_ · 0.5LiMO_2_ and reaction pathways during the first full cycle of 0.5Li_2_MnO_3_ · 0.5LiMn_0.50_Ni_0.35_Co_0.15_O_2_

**Figure 7:**
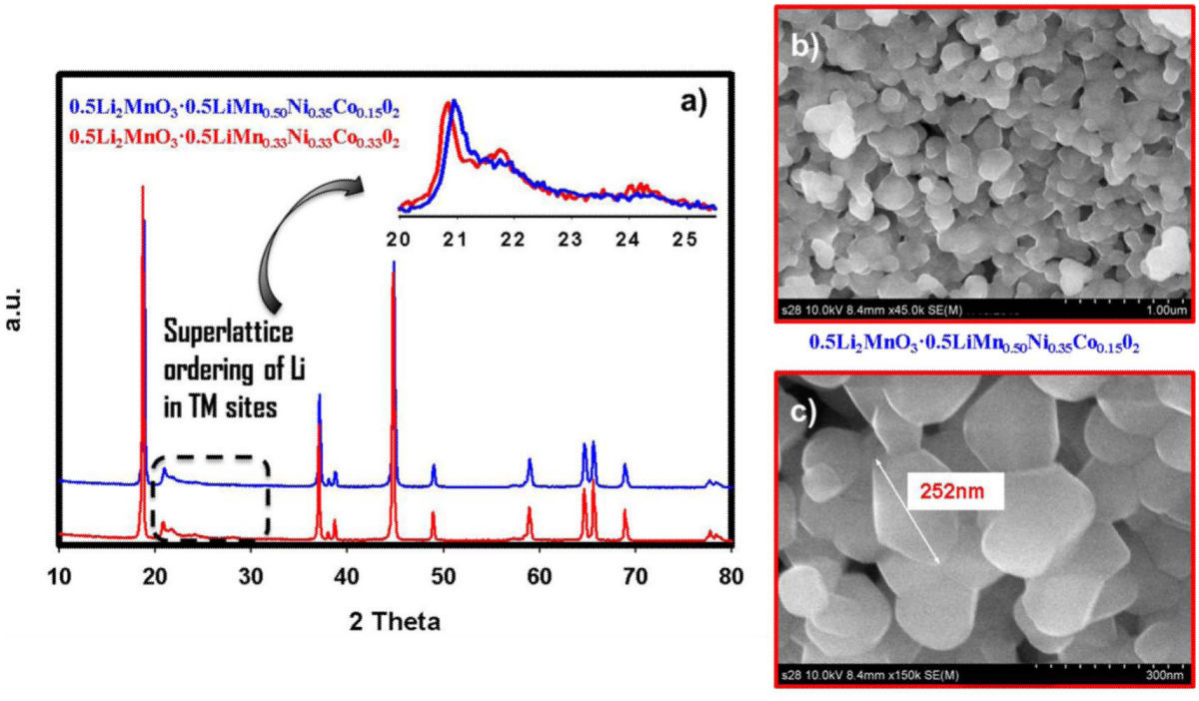
a-) XRD patterns of 0.5Li_2_MnO_3_ · 0.5LiMn_0.33_Ni_0.33_Co_0.33_O_2_ (red) and 0.5Li_2_MnO_3_ · 0.5LiMn_0.50_Ni_0.35_Co_0.15_O_2_ (blue). Inset Figure shows the magnified region for superlattice feature. b-) FESEM image of 0.5Li_2_MnO_3_ · 0.5LiMn_0.50_Ni_0.35_Co_0.15_O_2_ particles c-) the magnified FESEM image of 0.5Li_2_MnO_3_ · 0.5LiMn_0.50_Ni_0.35_Co_0.15_O_2_ showing the nanosized particles.

**Figure 8:**
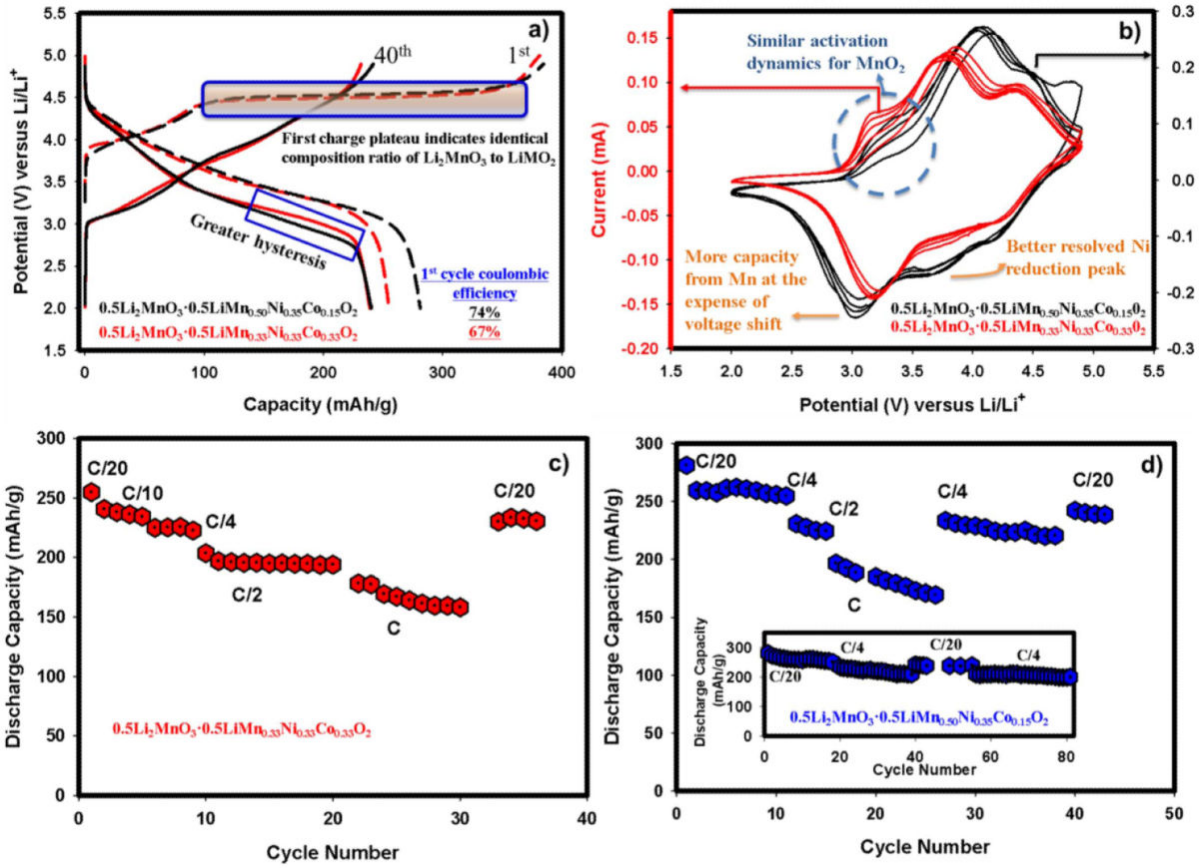
a-) The 1^st^ and 40^th^ charge and discharge profiles for both compounds run with C/20 rate between 2 and 4.9 V ranges b-) CV results run at the sweep rate of 50 μV/s for both materials showing redox behavior for each material. Cycling at a series of rates room temperature for c-) 0.5Li_2_MnO_3_0.5LiMn_0.33_Ni_0.33_Co_0.33_O_2_ d-) 0.5Li_2_MnO_3_ · 0.5LiMn_0.50_Ni_0.35_Co_0.15_O_2_. Inset in Figure (d) shows the cycling ability of 0.5Li_2_MnO_3_ · 0.5LiMn_0.50_Ni_0.35_Co_0.15_O_2_ at C/4 rate.

We varied the amount of transition metal in the LiMO_2_ portion of the material where x = 0.5 in order to determine the composite with the best electrochemical performance. The XRD patterns, shown in Figure 7a, for the two compounds 0.5Li_2_MnO_3_ · 0.5LiMn_0.33_Ni_0.33_Co_0.33_O_2_ (red) and 0.5Li_2_MnO_3_ · 0.5LiMn_0.50_Ni_0.35_Co_0.15_O_2_ (blue) resemble each other. The peak intensities and shapes, located between 20 and 25 2θ degrees, displayed in the inset of Figure 7a, are identical indicating that they both have the same amount of Li_2_MnO_3_. However, the material having lower amount of Mn and higher amount of Co in the LiMO_2_ segment has shown a slight shift toward smaller 2θ angles suggesting lattice expansion. This is expected if one considers the fact that Co has a larger radius than Mn that would cause lattice swelling. The FESEM images of 0.5Li_2_MnO_3_ · 0.5LiMn_0.50_Ni_0.35_Co_0.15_O_2_ at both low and high magnifications are presented in Figure 7b and 7c, respectively. From these Figures, it is clear that the material particles are approximately 200–250 nm and are agglomerated into larger particles creating an inner connected network that is beneficial to maintain capacity during cycling. The 1^st^ and 40^th^ charge and discharge voltage-capacity profiles for each compound are depicted in Figure 8a. An identical charge profile, specifically after 4.4 V, indicates both materials have the same amount of Li_2_MnO_3_ which further supports the conclusion drawn from XRD. This, in turn, enhanced the coulombic efficiency of 0.5Li_2_MnO_3_ · 0.5LiMn_0.50_Ni_0.35_Co_0.15_O_2_ because it has higher discharge capacity due to perhaps higher Mn content in the LiMO_2_ segment. At 40^th^ cycle, 0.5Li_2_MnO_3_ · 0.5LiMn_0.50_Ni_0.35_Co_0.15_O_2_ showed a slightly higher degree of voltage hysteresis due perhaps to the higher amount of Mn in the LiMO_2_ segment. Such fade is clear below 3 V in Figure 8a which is due to the Mn participation during the discharge reaction. These results were further complemented by the CV data. CV studies were performed in order to obtain further insight in to the redox behavior as well as potential shifts over the course of cycling. Figure 8b portrays the cyclic voltammograms of 0.5Li_2_MnO_3_ · 0.5LiMn_0.50_Ni_0.35_Co_0.15_O_2_ and 0.5Li_2_MnO_3_ · 0.5LiMn_0.33_Ni_0.33_Co_0.33_O_2_. The first oxidation peak at around 3.2 V is due to Mn oxidation associated with the MnO_2_ generated after the activation of Li_2_MnO_3_ in the first charge and its peak intensity become more pronounced as cycling continues indicating that its activity improves with cycling. Both materials exhibit very similar behavior during the first activation process since both have identical amount of Li_2_MnO_3_ as discerned from the plateau at 4.6 V in Figure 8a. However, one of the distinguishable traits is that Ni oxidation in 0.5Li_2_MnO_3_ · 0.5LiMn_0.50_Ni_0.35_Co_0.15_O_2_ shifts to higher potentials (~4.1 V) than that of 0.5Li_2_MnO_3_ · 0.5LiMn_0.33_Ni_0.33_Co_0.33_O_2_ (~3.8 V). This is a compelling finding in addition to the conclusion we had back in Figure 3 where we found a direct effect of Li_2_MnO_3_ on Ni oxidation process. In Figure 8b, however, both compounds have the same amount of Li_2_MnO_3_ except that for 0.5Li_2_MnO_3_ · 0.5LiMn_0.5_Ni_0.35_Co_0.15_O_2_ the amount of Mn is higher in the LiMO_2_ segment. This proves that not only the amount of Li_2_MnO_3_ in the composite influences Ni oxidation but also the ratio of the transition metals in LiMO_2_ portion affects its oxidation process. The higher Mn content in LiMO_2_ shifted the oxidation potential of Ni to above 4 V. During further oxidation, Co is being oxidized at similar potentials around 4.5 V for both materials. During reduction, Co is first reduced followed by Ni and finally Mn. Although Co reduction occurs at the same potential, Ni and Mn reduction behaviors are different for the two materials. For 0.5Li_2_MnO_3_ · 0.5LiMn_0.50_Ni_0.35_Co_0.15_O_2_, Ni reduction in the first cycle occurs around the same potential as 0.5Li_2_MnO_3_ · 0.5LiMn_0.33_Ni_0.33_Co_0.33_O_2_ which is at approximately 3.7 V. Nevertheless, for 0.5Li_2_MnO_3_ · 0.5LiMn_0.33_Ni_0.33_Co_0.33_O_2_, the same peak disappears after 2 cycles disabling the capacity contribution from Ni reduction at least in this potential. Thus, by preserving stable Ni reduction process 0.5Li_2_MnO_3_ · 0.5LiMn_0.50_Ni_0.35_Co_0.15_O_2_ has higher capacities as evident from the Figure 8a and 8b. The higher capacity, however, is compromised by voltage hysteresis as can be seen from the voltage shift to lower potentials in Figure 8b corresponding to Mn reduction. This shift, for 0.5Li_2_MnO_3_ · 0.5LiMn_0.50_Ni_0.35_Co_0.15_O_2_, is higher than that of 0.5Li_2_MnO_3_ · 0.5LiMn_0.33_Ni_0.33_Co_0.33_O_2_ due to greater amount of Mn in LiMO_2_ segment of composite structure. This further supports the result of galvanostatic data presented in figure 8a.We previously established a relationship between the voltage fade phenomenon and Mn existence in the LiMO_2_ phase^9^and concluded this as being primarily responsible for the transformation to the spinel phase as had been reported.^15^ Rate capability studies during the course of cycling for both materials are displayed in Figure 8c and 8d. It is obvious from these figures that both materials experienced relatively sharp capacity fade at high rates (i.e. 1 C). However at low rates (e.g. C/4, C/2) 0.5Li_2_MnO_3_ · 0.5LiMn_0.50_Ni_0.35_Co_0.15_O_2_ showed higher discharge capacities as plotted in Figure 8d. Cycle life assessment of 0.5Li_2_MnO_3_ · 0.5LiMn_0.50_Ni_0.35_Co_0.15_O_2_ at C/4 rate revealed that it can deliver a capacity of 200 mAh/g even after 80 cycles as shown in the inset of Figure 8d.

**Figure 9:**
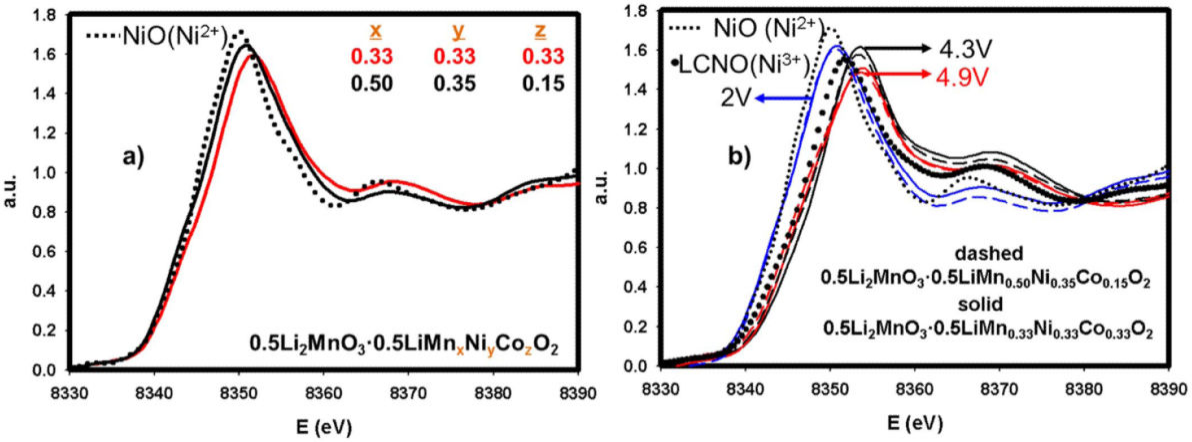
a-) XANES profiles for pristine 0.5Li_2_MnO_3_ · 0.5LiMn_0.33_Ni_0.33_Co_0.33_O_2_ (red) and 0.5Li_2_MnO_3_ · 0.5LiMn_0.50_Ni_0.35_Co_0.15_O_2_ (black) at Ni-K edge with Ni^2+^ reference compound. b-) XANES profiles at Ni-K edge for the same materials after charging up to 4.3 V and 4.9 V and discharged to 2 V along with Ni^2+^ and Ni^3+^references.

**Figure 10:**
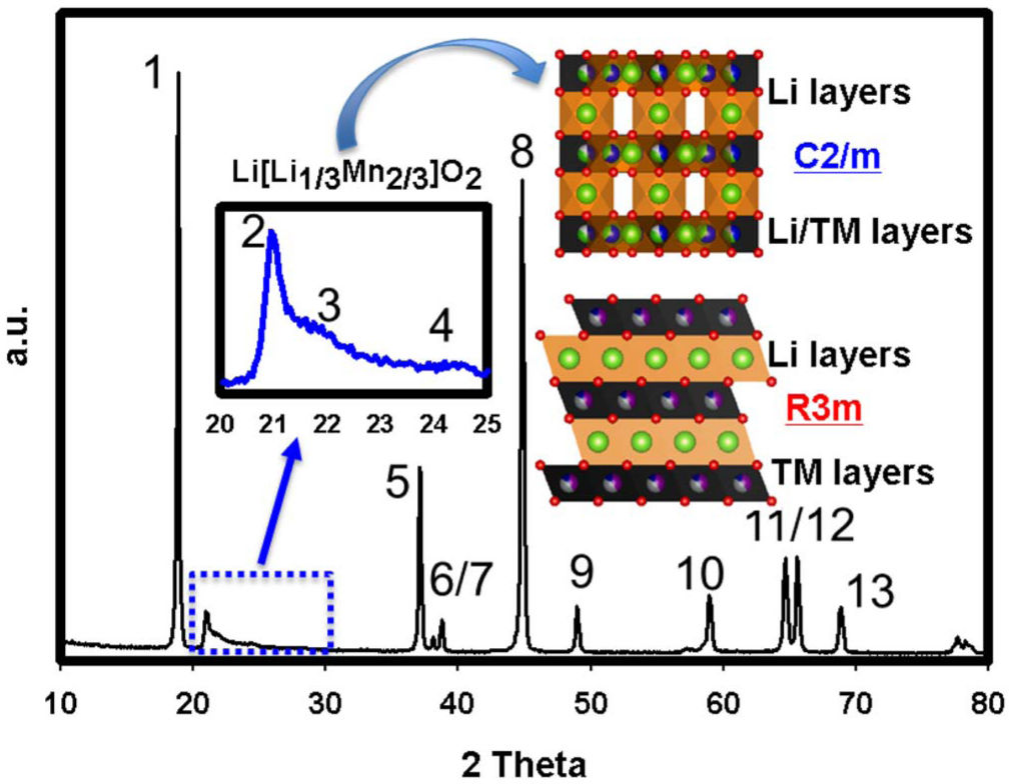
XRD patterns of 0.5Li_2_MnO_3_ · 0.5LiMn_0.50_Ni_0.35_Co_0.15_O_2_ (LLMNC) along with the unit cells for each phase. Peaks are numbered and explained in Table II.

We have executed XAS experiments on the Ni-K edge in order to determine the valence state and chemical environment of Ni atom in both materials. We first obtained Ni K-edge data on pristine materials along with NiO reference as depicted in Figure 9a. The shape of the XANES profiles for each compound appears to be similar suggesting the same chemical environment at the Ni absorber atom. The valence states of Ni in each material, however, are different. This can be explained through the neutrality rule for a metal oxide. If one increases the amount of Mn, which has higher valence state (4^+^) than Ni (2^+^) in the pristine states, it is plausible to expect that the valence state of Ni should decrease to balance the negative charge on the oxide. In our case, 0.5Li_2_MnO_3_ · 0.5LiMn_0.50_Ni_0.35_Co_0.15_O_2_ has higher amount of Mn in the LiMO_2_ segment than in 0.5Li_2_MnO_3_ · 0.5LiMn_0.33_Ni_0.33_Co_0.33_O_2_. This results in a lower valence state for Ni close to Ni^2+^ in 0.5Li_2_MnO_3_ · 0.5LiMn_0.50_Ni_0.35_Co_0.15_O_2_. Additionally, we performed XAS experiments during the first full cycle at different potential intervals in order to compare possible structural and valence state changes. Figure 9b displays XANES profiles during the first full cycle at different potentials along with Ni^2+^ and Ni^3+^ references. During charging, it is clear that both materials have identical valence states at each potential. Indeed, after 4.3 V no oxidation was observed for Ni, suggesting that complete oxidation of Ni takes place at or before 4.3 V for both compounds. However, during the first discharge to 2 V a critical change was observed. While the pristine materials, displayed in figure 9a, have different Ni valence states, at 2 V they possess identical Ni oxidation states. This is most likely due to the integration of Mn atom into the regular layered structure after the activation of Li_2_MnO_3_. This redox participation after the first charge leads to a decrease of the valence state of Mn in both materials inducing the same Ni valence state at 2 V. In order to obtain a deeper understanding of the reaction mechanisms in Li rich layered metal oxides during charge/discharge cycling, we have used 0.5Li_2_MnO_3_ · 0.5LiMn_0.50_Ni_0.35_Co_0.15_O_2_ (LLMNC) which alternatively can be written as Li_1.2_Mn_0.6_Ni_0.14_Co_0.06_O_2_, as an example. In academia, similar materials also have been used widely among other lithium rich candidates.^5,7,29–32^ Figure 10 shows the XRD pattern of as-synthesized LLMNC pristine powder along with the unit cells visualizing corresponding to the C2/m space group where some of the Li resides in TM sites and the R3m space group in which alternating Li and TM layers are periodically stacked. Each peak is numbered and their corresponding space groups are listed in Table II. The peaks numbered as 2, 3 and 4, arising from Li existence in TM sites, are clearly evident between 20–25 2θ degrees. These peaks cannot be indexed to R3m space group and solely matches with the C2/m space group pattern. Therefore, all peaks in the pristine material can only be indexed to C2/m, although a consensus as to whether the Li rich layered metal oxides are a composite of Li_2_MnO_3_ and LiMO_2_ or a solid solution^18,33^of LiMO_2_ in Li_2_MnO_3_ with C2/m phase is still not achieved. Nonetheless, the widely held notion of a composite structure^21,23,28,34,35^ together with our previous observations^9,10^ lead us to see this class of cathode materials as composite structures having two phases that are perfectly integrated at the atomic level.

Identifying the irreversible capacity loss (ICL) for Li rich composite oxides materials is crucial since it affects the long term voltage fade and cycling stability. Although materials having higher ICL results in higher 1^st^discharge capacities, they showed a higher degree of voltage hysteresis as well as capacity loss during cycling. ICL calculations are often performed for capacities at low current densities, i.e. low C rates, in order to realize a fully reversible reaction. Therefore, it is vital to determine the theoretical capacity of a given lithium rich compound which influences the C rate calculations. In order to calculate theoretical specific capacities of a Li rich material, in this case LLMNC, we used the equation 1 where Q signifies theoretical specific capacity (mAh/g), N is equal to the number of electrons involved in the reaction, F is the Faraday’s constant (96485 C/mole or 26.801 Ah/mole) and M is the molecular weight of the cathode (g/mole).

[1] $$Q\;=\;\frac{N\;F}{M}$$

**Figure 11:**
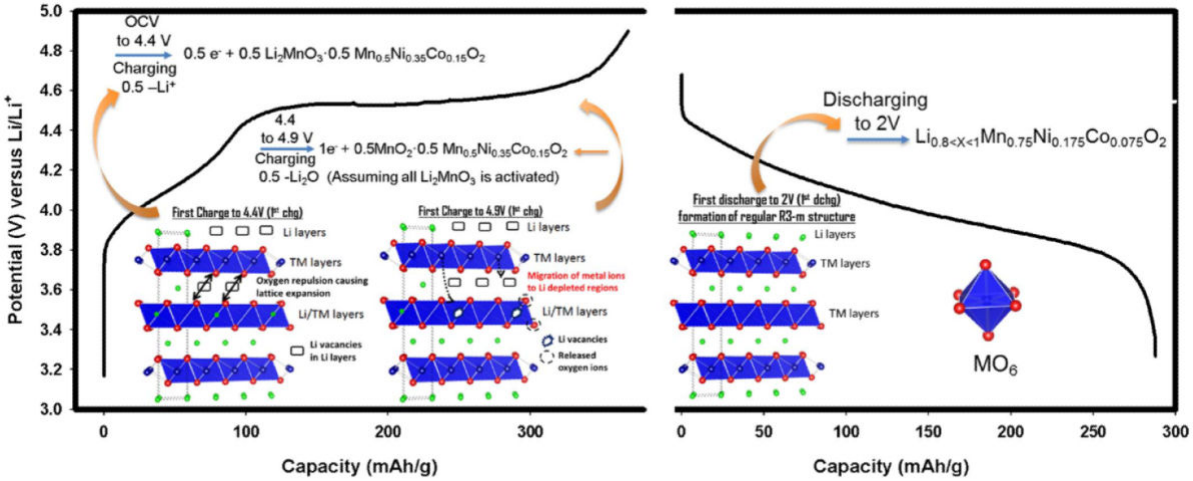
The first charge and discharge profiles for 0.5Li_2_MnO_3_ · 0.5LiMn_0.50_Ni_0.35_Co_0.15_O_2_ (LLMNC). Crystallographic representation was shown for 4.4 V, 4.9 V and 2 V regions unraveling reaction paths.

First of all, the theoretical specific capacities of Li_2_MnO_3_ and LiMn_0.5_Ni_0.35_Co_0.15_O_2_ are 458 mAh/g and 279 mAh/g, respectively. Because half a mole each of these compounds is present in the LLMNC material, the overall theoretical capacity for 0.5Li_2_MnO_3_ · 0.5LiMn_0.50_Ni_0.35_Co_0.15_O_2_ is expected to be around 370 mAh/g. Although, as can be seen in Figure 11, the capacity obtained from the first charge augurs well with the theoretical capacity we calculated, we are still not ruling out some capacity contribution from the electrolyte oxidation at high potentials above 4.6 V. This is plausible if one considers that charging Li_2_MnO_3_ even up to 5 V extracts only 85% of Li present in it.^27^ During the first discharge, the material delivered a discharge capacity of approximately 280 mAh/g. This high ICL is evidently caused mostly by the irreversible Li extraction from Li_2_MnO_3_. Previously, we^36^ and others have shown^5,11^ that superlattice feature is vanished after the 1^st^ full cycle. Therefore, the discharge product could have the Li_0.8*<*x*<*1_MO_2_ (M = TM) structure, due to perfect compatibility of MnO_2_ (originating from Li_2_MnO_3_) and LiMO_2_, to host Li. However valence state of each TM plays a significant role in hosting the quantity of Li. For example, if one assumes the final TM oxidation states of Mn, Ni and Co after the first discharge at 2 V as 3.5^+^, 2^+^ and 3^+^, respectively, then the Mn_0.75_Ni_0.175_Co_0.075_O_2_ structure can only host 0.8Li. This ambiguity we believe can be enlightened by using XAS which is an excellent tool to fingerprint the oxidation state of a transition metal during electrochemical reactions. Reaction paths during each potential intervals are illustrated in the Figure 11. As discussed above with XAS data, during charging until 4.4 V the only transition metals that are being oxidized are Ni and Co, albeit slight oxidation for Co might occur after 4.4 V. This whole oxidation process is accompanied by Li removal from Li layers residing in the LiMO_2_ phase. Li extraction from LiMO_2_ causes oxygen repulsion which leads to lattice expansion. After 4.4 V where the activation of Li_2_MnO_3_ phase begins, delithiation takes place in both phases which cannot be differentiated. During this step, some of the transition metals especially Mn and possibly Co tend to occupy Li depleted regions thereby creating structural rearrangements. The possible oxidation of electrolyte takes place at high potentials^37^ but identifying such oxidation process and its reversibility is beyond the scope of this paper. Some of the early reports showed that O_2_ can be reduced to superoxide^5^ during discharge below 3 V and is consumed by side product formation such as Li_2_CO_3_. After successful removal of all possible Li ions from both phases, the composite structure behaves like a regular LiMO_2_ structure having R3m space group during the first discharge to 2 V.

### Enhancement of rate capabilities and cycling stabilities

**Figure 12:**
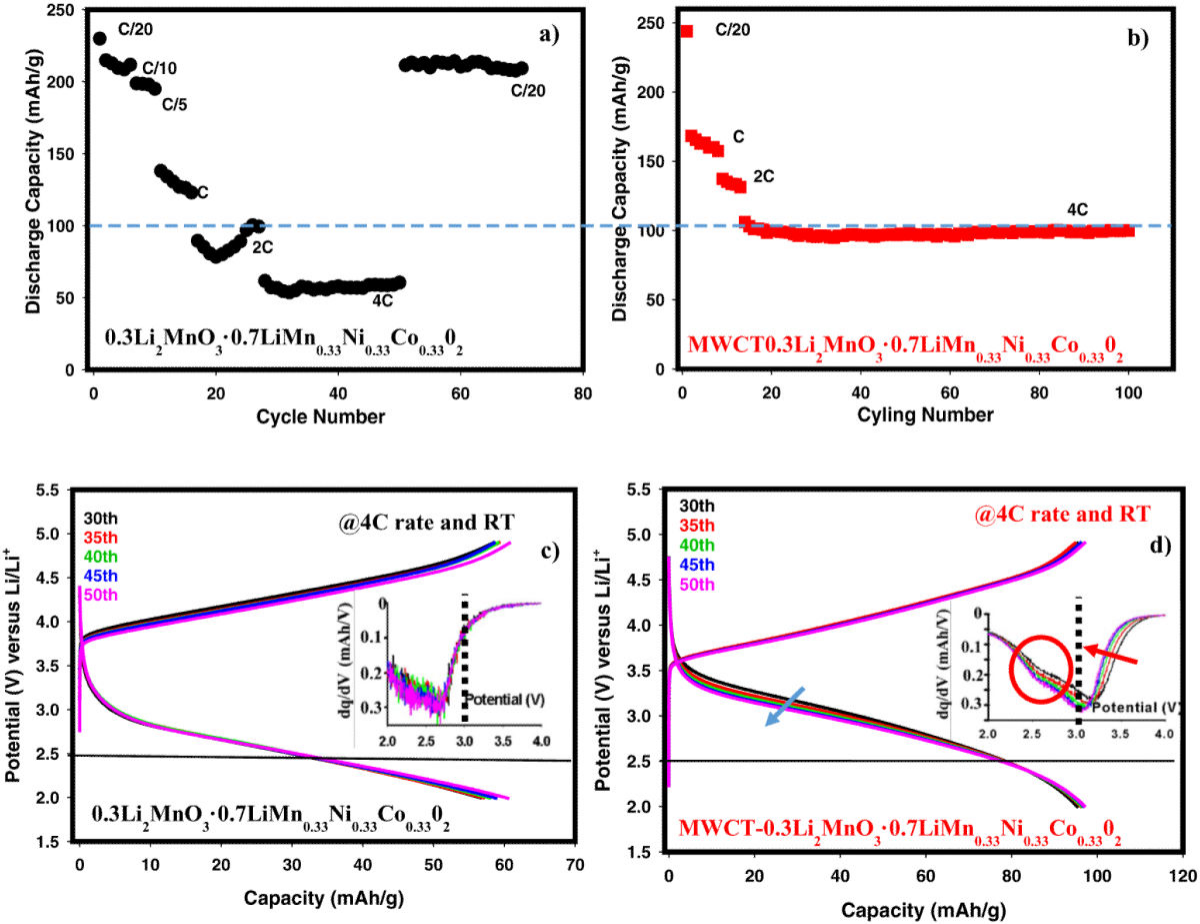
Cycling and rate capabilities of a-) 0.3Li_2_MnO_3_ · 0.7LiMn_0.33_Ni_0.33_Co_0.33_O_2_. b-) MWCT (2.5 wt% instead of Super P) mixed 0.3Li_2_MnO_3_ · 0.7LiMn_0.33_Ni_0.33_Co_0.33_O_2_. Charge discharge profiles and differential capacity plots for c-) 0.3Li_2_MnO_3_ · 0.7LiMn_0.33_Ni_0.33_Co_0.33_O_2_. d-) MWCT (2.5 wt% in place of Super P) mixed 0.3Li_2_MnO_3_ · 0.7LiMn_0.33_Ni_0.33_Co_0.33_O_2_.

The various Li rich materials discussed above did not demonstrate high rate capabilities and good cycling stability. One of the factors contributing to this is the low electronic conductivity of these materials. We have used a simple doping strategy to obtain high rate capabilities (>1 *C*) with excellent stability for over hundred cycles. We used a small amount (2.5 wt% of Super P used in the electrode) of multiwall carbon nanotube (MWCNT) in place of Super P carbon in the electrode to boost the rate capabilities of 0.3Li_2_MnO_3_ · 0.7LiMn_0.33_Ni_0.33_Co_0.33_O_2_ which has good capacity retention at the C/20 rate as shown in Figure 12a. The same material, however, shows only a capacity of 55 mAh/g at the 4C rate. Mixing 2.5 wt% of MWCT in the electrode instead of Super P during the preparation of cathode enhanced the capacity at 4*C* rate by almost 100% reaching 100 mAh/g without any capacity fade for over hundred cycles as displayed in Figure 12b. It is very well known that MWCTs have high electronic and ionic conductivities^38^ which appear to play a role here to increase the rate capability of this composite cathode material. Charge-discharge voltage versus capacity profiles between the 30^th^ to 50^th^ cycles are plotted in Figure 12c and 12d. One can easily recognize from Figure 12d that the cathode with MWCT shows higher capacity above 2.5 V whilst a small voltage decay is clear above 3 V. In order to ascertain why MWCT enhanced the specific capacities obtained at the 4C rate, differential capacity plots from the same data presented in Figure 12c and 12d were extracted and displayed as insets of these figures. A very first distinct feature for MWCT mixed cathode, shown in the inset of Figure 12d, is that it has well-resolved redox peaks. During discharge, the peaks around 3 V and 2.5 V corresponding to Ni/Co and Mn reduction, respectively, are clearly observed. Thus MWCNT improves transition metal participation during reaction probably due to the higher conductivity of the electrode. In contrast to this performance, the same compound without MWCT, displayed in Figure 12a and 12c, shows neither a reasonable capacity nor resolved peaks pertaining to Ni/Co reduction above 3 V. In addition, a clearer Mn reduction in the inset of Figure 12d, shown in oval shape, resulted in a form of voltage hysteresis exhibited in Figure 12d. From these results, we can conclude that enhancement of rate capabilities through MWCT does not have any ramifications on voltage fade phenomenon rather it triggers Mn reduction which causes voltage swelling.

## Conclusions

We carried out a comprehensive investigation of Li rich layered metal oxide cathode materials for Li-ion batteries with all of them synthesized under identical conditions combined with detailed structural and electrochemical characterization. This has resulted in the selection of an optimum cathode material of this class for Li-ion batteries. We have found that the highest Li_2_MnO_3_-containing composite cathode in the series xLi_2_MnO_3_ · (1-x)LiMn_0.33_Ni_0.33_Co_0.33_O_2_ in which x = 0.1, 0.2, 0.3, 0.5 or 0.7 demonstrated greater voltage fade with cycling. Ultimately this causes low energy and power densities when these materials are used in Li-ion cells. Lower amounts of Li_2_MnO_3_ incorporated into Li-rich composite oxides delivered low capacities despite their low voltage hysteresis. Among the various materials studied the general composition 0.5Li_2_MnO_3_ · 0.5LiMO_2_ was found to be the optimum in terms of both specific capacity and overall energy output. In this composite oxide, the amount of Mn located in the LiMO_2_ portion played a crucial role in improving its specific capacity. Thus we synthesized and characterized 0.5Li_2_MnO_3_ · 0.5LiMn_0.50_Ni_0.35_Co_0.15_O_2_ as the best material in terms of electrochemical performance in Li cells. The addition of a small amount of MWCT to the electrode was found to be effective in increasing the rate capability and cycling stability of these cathode materials due to the higher electronic conductivity of the electrode.

## Figures and Tables

**Table I:** First charge and discharge capacities of xLi_2_MnO_3_ · (1-x) LiMn_0.33_Ni_0.33_Co_0.33_O_2_ (0.1≤x≤0.7) at C/20 rate between 2 and 4.9 V at room temperature.

Cathodes xLi_2_MnO_3_.(l-x) LiMn_0.33_Ni_0.33_Co_0.33_O_2_	1^st^ Charge capacity (mAh/g)	1^st^ Discharge capacity (mAh/g)
X = 0.1	316.26	224.43
X = 0.2	324.57	245.35
X = 0.3	340.87	244.31
X = 0.5	375.62	254.82
X = 0.7	352.82	233.57

**Table II:** Plane identifications for each phases presented in Figure 10. Numbered peaks were shown in Figure 10.

Peak Number	Li_2_MnO_3_ C2/m planes	LiMnO_2_ R3m planes
1	(001)	(003)
2	(020)	–
3	(110)	–
4	(−111)	–
5	(−201)	(101)
6	(−112)	(006)
7	(−131)	(012)
8	(131)	(104)
9	(−132)	(015)
10	(132)	(107)
11	(−133)	(018)
12	(−331)	(110)
13	(330)	(113)
